# Testing the *FMR1* Promoter for Mosaicism in DNA Methylation among CpG Sites, Strands, and Cells in *FMR1*-Expressing Males with Fragile X Syndrome

**DOI:** 10.1371/journal.pone.0023648

**Published:** 2011-08-31

**Authors:** Reinhard Stöger, Diane P. Genereux, Randi J. Hagerman, Paul J. Hagerman, Flora Tassone, Charles D. Laird

**Affiliations:** 1 Department of Biology, University of Washington, Seattle, Washington, United States of America; 2 Department of Genome Sciences, University of Washington, Seattle, Washington, United States of America; 3 Medical Investigation of Neurodevelopmental Disorders Institute, University of California Davis Medical Center, Sacramento, California, United States of America; 4 Department of Pediatrics, School of Medicine, University of California Davis, Davis, California, United States of America; 5 Department of Biochemistry and Molecular Medicine, School of Medicine, University of California Davis, Davis, California, United States of America; 6 School of Biosciences, University of Nottingham, Leicestershire, United Kingdom; Bellvitge Biomedical Research Institute (IDIBELL), Spain

## Abstract

Variability among individuals in the severity of fragile X syndrome (FXS) is influenced by epigenetic methylation mosaicism, which may also be common in other complex disorders. The epigenetic signal of dense promoter DNA methylation is usually associated with gene silencing, as was initially reported for *FMR1* alleles in individuals with FXS. A paradox arose when significant levels of *FMR1* mRNA were reported for some males with FXS who had been reported to have predominately methylated alleles. We have used hairpin-bisufite PCR, validated with molecular batch-stamps and barcodes, to collect and assess double-stranded DNA methylation patterns from these previously studied males. These patterns enable us to distinguish among three possible forms of methylation mosaicism, any one of which could explain *FMR1* expression in these males. Our data indicate that cryptic inter-cell mosaicism in DNA methylation can account for the presence of *FMR1* mRNA in some individuals with FXS.

## Introduction

Epigenetic mosaicism strongly influences the variable phenotypes characteristic of at least two neurodevelopmental disorders: fragile X syndrome (FXS) and Rett syndrome [1], [2], [3], [4], [5], [6]. FXS is characterized by a broad profile of impairment including intellectual disabilities and comorbidity with autism (see review [7]). Current frequency figures for FXS range from 1∶2500–8000 in females and 1∶4000 in males in the general population [8], [9], [10], [11].

The molecular epigenetic signature of FXS includes dense DNA methylation at the FMR1 promoter. Dense methylation of promoter regions is a common feature of silenced genes [12]. Such silencing was initially reported also to hold for abnormally methylated *FMR1* alleles in individuals with FXS [13]. In FXS, abnormal methylation can occur within the promoter region of alleles that contain an expanded CGG repeat [13]. The transcriptional status of *FMR1* is an important variable in the diagnosis and prognosis of FXS, and is especially informative because of frequent examples of inter-cell methylation mosaicism in samples routinely used for diagnosis [2], [3], . The most severely affected males are typically those whose alleles are densely methylated at this locus in all cells sampled; such males have typically been thought not to express *FMR1* mRNA. A paradox arose when Tassone and colleagues reported significant levels of *FMR1* mRNA in the majority of individuals from a cohort of males with FXS found to have methylated, full mutation *FMR1* alleles, and to lack subpopulations of premutation alleles [16]. These individuals showed no evidence of correspondence between levels of *FMR1* mRNA and the severity of the fragile X phenotype. Large CGG tracts in the 5′ region of mRNAs transcribed from full mutation *FMR1* alleles, and even in the premutation range, have been found to inhibit protein synthesis [17], [18]. Thus, even in the presence of an unmethylated, full mutation allele, these individuals do not express appreciable levels of *FMR1*-encoded protein, FMRP, and do not have phenotypes markedly different from those of males who lack *FMR1* mRNA.

Here, we address a more basic problem in molecular biology. Are certain types of DNA methylation patterns on heavily methylated promoters permissive of RNA transcription? One possible explanation for the unexpected findings of Tassone and colleagues is that clinical assays, and most research protocols that are designed to ascertain methylation status, including Southern hybridization, are not able to detect all possible types of methylation mosaicism. We reasoned that if methylation mosaicism were permissive for *FMR1* expression, then double-stranded DNA methylation patterns of the *FMR1* promoter might reveal mosaicism of an unusual form not previously assessed in FXS. Using hairpin-bisulfite PCR with batch-stamps and barcodes we searched for three possible types of mosaicism by determining patterns of cytosine methylation on the two complementary strands of individual DNA molecules, [19]. These methods provide authenticated information on double-stranded methylation patterns, and here enabled us to distinguish valid from contaminant and redundant sequences. The samples described by Tassone and colleagues thus provide an opportunity to apply new methods to distinguish among different kinds of mosaicism for DNA methylation. We collected double-stranded methylation patterns from DNA of nine males with full mutation alleles reported to be fully methylated, using a subset of the samples that Tassone and colleagues used in their 2001 study [16].

**Figure 1:**
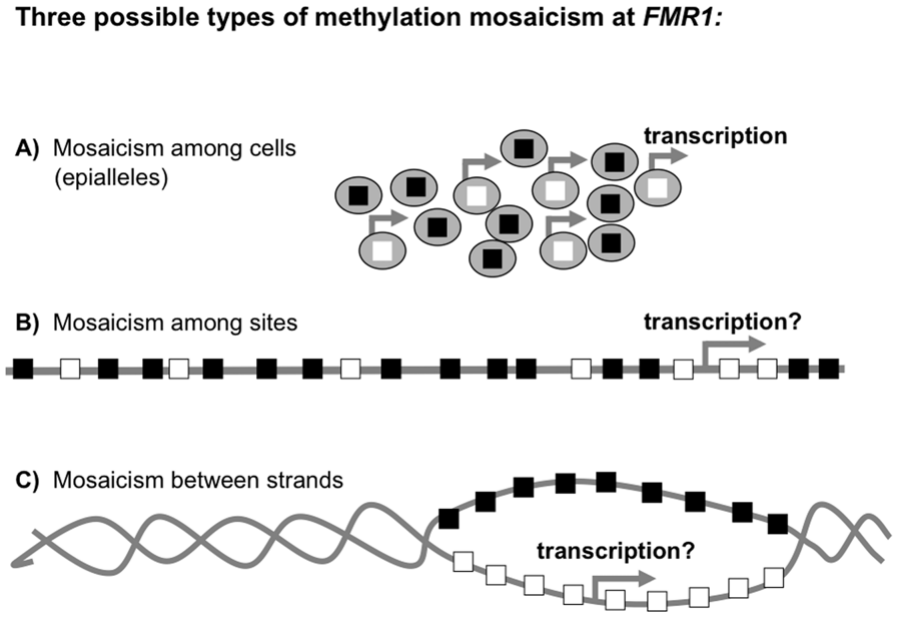
Three possible types of mosaicism for cytosine methylation at *FMR1*. (A) Mosaicism among cells, with either unmethylated (open squares) or hypermethylated (filled squares) epialleles; (B) mosaicism among CpG sites; and (C) mosaicism between the two strands of an individual DNA molecule. For the latter two possibilities, each square represents a CpG on a single strand of DNA; filled boxes depict methylated CpGs and open boxes depict unmethylated CpGs. Arrows represent possible transcriptional activity under each methylation-mosaicism scenario.

Here, we report results of our tests to distinguish among three possible types of methylation mosaicism: among cells of an individual (Figure 1A), at CpG sites within an allele (Figure 1B), between the two strands of an individual DNA molecule (Figure 1C).

## Results

DNA samples used for the Tassone et al. study [16] were originally evaluated using Southern hybridization analysis, which showed the presence of hyper-methylated alleles (>200 CGG repeats). The authors suggested that the *FMR1* mRNA transcription observed in those subjects could have derived from densely methylated promoters, in contrast to the typically silenced state of such methylated promoters [16], [12]. Due to the detection limits of the Southern hybridization approach, an alternative possibility is that these samples contained alleles with previously unexamined types of methylation mosaicism that could be permissive for *FMR1* transcription.

**Figure 2:**
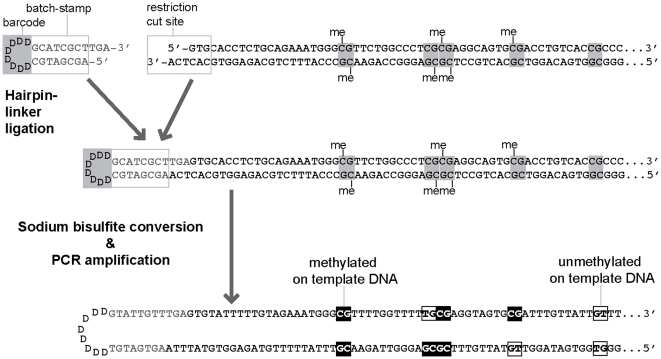
Hairpin–bisulfite PCR at *FMR1* (after Miner et al. [**20**]). The genomic sequence from both strands of the *FMR1* promoter, including the first five of the analyzed analyzed CpG sites, is shown. A hairpin linker, here illustrated for hairpin I, is ligated to both strands of an individual DNA molecule prior to bisulfite conversion and PCR amplification. The bisulfite-conversion reaction converts cytosine, but not 5-methylcytosine (“me”), to uracil, thus preserving information on methylation patterns in genomic DNA. After PCR amplification, uracil residues will appear as thymine; a cytosine detected in the sequence of a PCR product therefore indicates methylation of that base in the genomic DNA.

To address this issue, we have used hairpin-bisulfite PCR [19], validated with molecular batch-stamps and barcodes [20], to collect and assess authenticated, double-stranded DNA methylation patterns at *FMR1* in these previously studied males [16] (Figure 2). These methods here enabled us to distinguish valid from contaminant and redundant sequences, and thus to provide more quantitative information on possible methylation mosaicism.

**Figure 3:**
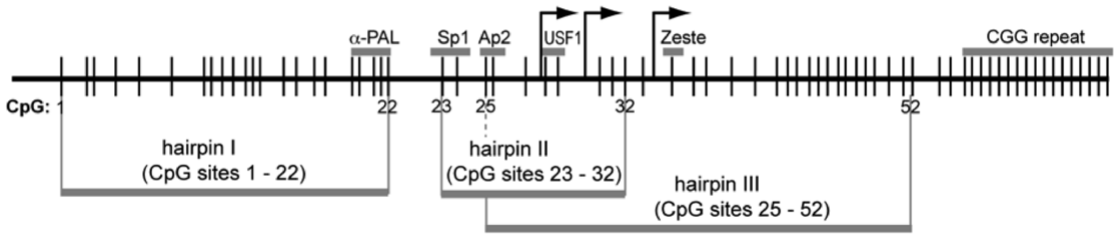
Three separate hairpin–bisulfite PCR assays for the *FMR1* promoter. Three hairpins were developed to analyze a total of 52 CpG dyads representing 104 CpGs on both strands of the *FMR1* promoter. Small vertical lines depict the distribution of CpG dyads present within the core region of the human *FMR1* promoter and the adjacent track of CGG repeats. Horizontal gray bars above the CpG plot indicate the location of binding sites for transcription factors and the position of the CGG repeat. Arrows depict three initiation sites for transcription of *FMR1*.

The core of the *FMR1* promoter resides within a ∼400-nucleotide sequence immediately upstream of the CGG repeat. This region contains 52 CpG dinucleotides, some of which overlap or flank transcription-factor binding elements [21], [22] (Figure 3). To examine these 52 CpG sites, we developed three separate hairpin-bisulfite PCR assays covering CpG sites 1–22 (hairpin I), CpG sites 23–32, (hairpin II), and CpG sites 25–52 (hairpin III) (Figure 3). Detailed results of the methylation analysis of the *FMR1* promoter region are reported in Table 1.

**Table 1:** Summary of *FMR1*-promoter methylation in samples from males with FXS and from female controls.

Individual	mRNA levels	hairpin assay I (sites 1–22)					hairpin assay II (sites 23–32)					hairpin assay III (sites 25–52)				
males with full mutation		epialleles analyzed	meth	un-meth	f meth alleles	average density	epialleles analyzed	meth	un-meth	f meth alleles	average density	epialleles analyzed	meth	un-meth	f meth alleles	average density
male 1	0.0	25	25	0	1.00	0.90						24	24	0	1.00	0.87
male 2	0.0						5	5	0	1.00	0.65	7	7	0	1.00	0.83
male 6	0.0											7	7	0	1.00	0.90
male 23	0.0						29	29	0	1.00	0.83	11	11	0	1.00	0.76
male 33	0.0											10	10	0	1.00	0.70
male 3	1.4	16	11	5	0.69	0.90										
male 20	1.0	17	8	9	0.47	0.89										
male 14	0.8	35	35	0	1.00	0.92	34	34	0	1.00	0.73	16	16	0	1.00	0.84
male 78	0.7						7	7	0	1.00	0.68					
**pooled data for all males with fragile X**		**93**	**79**	**14**		**0.91**	**75**	**75**	**0**		**0.76**	**75**	**75**	**0**		**0.83**

normal female controls		epialleles analyzed	meth	un-meth	f meth alleles	average density	epialleles analyzed	meth	un-meth	f meth alleles	average density	epialleles analyzed	meth	un-meth	f meth alleles	average density
normal female F1		58	31	27	0.53	0.83	32	30	2	0.94	0.67					
normal female F2		28	15	13	0.54	0.85										
normal female F3		78	33	45	0.42	0.87										
normal female cF3		63	32	31	0.51	0.85						22	16	6	0.73	0.72
normal female cF4		67	30	37	0.45	0.83										
normal female cF7		58	20	38	0.34	0.86	28	25	3	0.89	0.57	2	1	1	0.50	0.59
normal female F7		35	14	21	0.40	0.89										
normal female cF9							7	7	0	1.00	0.57					
normal female cF10							11	10	1	0.91	0.60					
normal female cF11												5	2	3	0.40	0.75
normal female cF5												24	20	4	0.83	0.63
normal female A68												30	16	14	0.53	0.69
**pooled data for normal females**		**387**	**175**	**212**	**0.45**	**0.85**	**78**	**72**	**6**	**0.92**	**0.62**	**83**	**55**	**28**	**0.66**	**0.67**

Each of the three hairpin assays was monitored for biased amplification of either methylated or unmethylated epialleles using pooled data obtained from normal-female controls. The relative *FMR1* mRNA levels for males with FXS have been reported previously [16]. The columns labeled “f meth alleles” give the observed frequencies of methylated epialleles.

### Methylation mosaicism among CpG sites

Using the three hairpin assays described above, we obtained methylation-information for a total of 243 double-stranded DNA molecules, here termed “epialleles”, from males with FXS (Table 1). Of these molecules, 229 represented methylated epialleles, while only 14 represented unmethylated epialleles.

**Figure 4:**
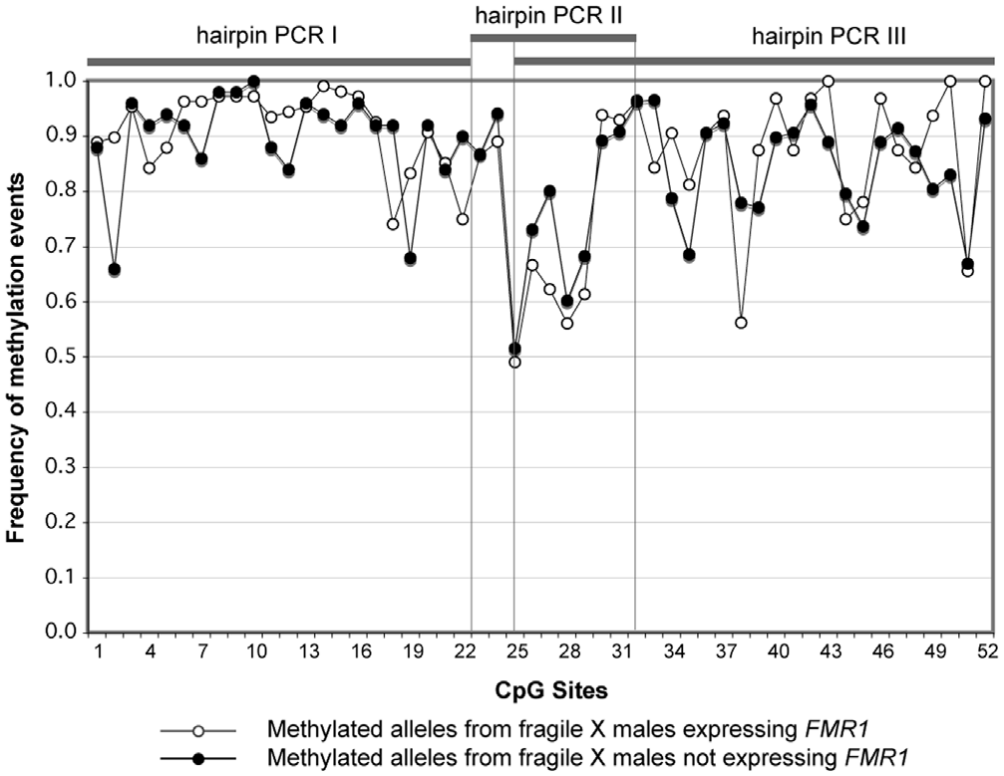
CpG site-specific methylation frequencies among hypermethylated epialleles. CpG site-specific methylation frequencies among hypermethylated epialleles as observed in samples from males with FXS who express (open circles), or do not express (filled circles) *FMR1*. Data were pooled across hairpin assays I, II, and III. Vertical lines indicate the boundaries of the three different hairpins.

These double-stranded data are quantitatively consistent with our earlier single-stranded methylation data [3]. For the methylated epialleles, we observed extensive variation in methylation frequencies among CpG sites. Site-specific frequencies ranged from 0.5 (site 25) to 1.0 (site 10; Figure 4), similar to the variation described previously for some regions of *FMR1* [3]. The mean frequency of methylation calculated here from both strands at CpG sites 1–22 in the males with FXS (0.91; Table 1) is also similar to our earlier estimates for the top-strand-only data for males with FXS (0.95) [3]. Our data for methylated, inactive-X alleles from normal females, reported here, (0.85; Table 1), are comparable to our previously published results (0.87) [3]. Thus, the double-stranded and single-stranded methylation data are in good agreement for these parameters.

To test for consistency of double-stranded methylation data collected using different hairpin protocols, we compared data at CpG sites 25 to 32, as ascertained using hairpins II and III, which overlapped for these sites. For this comparison, we focused on samples for which assays with both hairpin II and III were successful: non-expressing males #2, #23, and female #cF7 (Table 1). The two hairpin assays gave concordant results for all three individuals regarding inter-cell mosaicism. Male samples #2 and #23 showed only methylated epialleles, and the female control sample #cF7 showed a mixture of methylated and unmethylated epialleles, as would be predicted for a female having one active- and one inactive-X chromosome in each cell.

A comparison of site-specific methylation densities for male #2 and female #cF7 at these eight CpG sites yielded average densities of methylation that did not differ significantly (p>0.064, 1 d.f., χ_2_ = 3.4; p>0.4, 1 d.f., χ_2_ = 0.5, respectively). For the third individual, male #23, there was a significant difference in average methylation densities as estimated using hairpins II and III (p<0.0001, 1 d.f., χ_2_ = 15.8). This p-value is more than ten times smaller than the significance limit of 0.0015 that is recommended after the conservative Bonferroni correction for multiple comparisons, in this case for the three individuals. We have no explanation for this significant difference in estimates. In all other respects, hairpins II and III gave concordant results for this individual. Unfortunately we were not successful in using both hairpins on samples from any other individual of this cohort.

Methylation densities obtained with the three different hairpins and averaged over the 52 analyzed CpG dyads on methylated epialleles were virtually indistinguishable between *FMR1* expressing and non-expressing males (0.830 vs. 0.824, respectively). To further explore possible variation among sites, we next performed an analysis of variance (ANOVA) for data sets obtained with the three different hairpins. For hairpin I, there was no significant effect of *FMR1* expression status (p>0.37), or individual (p>0.28). For hairpin III, the ANOVA indicated no significant effect of expression status (p>0.1), but a very significant effect of individual within the group of non-expressing males (p<0.00001). Thus, we conclude that this variation in methylation densities is not related to *FMR1* expression but is instead due to variation among individuals with an inactive *FMR1* allele. We have also seen variation in methylation densities in the control dataset from normal females, reflecting density of methylation on the inactive-X chromosome (Table 1).

The analysis for hairpin II was potentially interesting in that it revealed significant effects of expression group (p<0.01), and individual (p<0.03). We sought, but did not find, a subset of methylated sequences that had methylation densities low enough to permit transcription. The subset of methylated epialleles with the lowest densities of methylation in this region, identified as those with ≤13 of 20 CpGs (considering both strands), averaged 0.55 and 0.60 from expressing (34% of sequences) and non-expressing males (18% of sequences), respectively. There was no significant difference in methylation density between the two groups (p>0.5, 1 d.f., χ_2_ = 0.39). Thus, there was no evidence for the existence of a subgroup of alleles with overall low-density methylation that might distinguish expressing from non-expressing males.

Of the ten CpG sites assayed by hairpin II (CpG sites 23–32), six are located within binding sites for transcription factors Sp1, Ap2 and USF1 (Figure 3), underscoring their potential relevance to *FMR1* expression. None of these six CpG sites, as ascertained by hairpin II, had methylation densities that differed significantly between expressing and non-expressing groups.

CpG dyad 27, which does not overlap with a transcription-factor binding motif, was the only site assayed with hairpin II that showed a significant difference in methylation frequency between expressing and non-expressing males (0.52 and 0.76, respectively; p<0.02 after Bonferroni correction for multiple comparisons). However, the subset of low-methylation-density alleles, which would be most likely to express *FMR1*, showed no significant difference between expressing and non-expressing males in methylation frequency for CpG site 27 (0.29 versus 0.50, p<0.2); this p-value is well above the 0.005 required for significance with a Bonferroni correction for multiple comparisons. In addition, analysis of data obtained with hairpin III, which also assays methylation at CpG site 27, provides no evidence for a pivotal role of this site in *FMR1* expression (p≤0.48).

In addition, the lack of methylation at site 27 is unlikely to be sufficient for transcription because an average of 20% of alleles from non-expressing males sampled in this study have this site unmethylated. Were this lack of methylation sufficient for transcription, males with FXS who exhibit this type of site-specific mosaicism would have *FMR1* mRNA at levels greater than 1.4, assuming a seven-fold average level of elevated transcription for unmethylated, expanded alleles that approach the size of full mutation alleles [23]. The limit of detection of *FMR1* mRNA – less than 0.01 of normal cells [16] – is substantially below such levels, establishing that an unmethylated CpG at site 27 of an individual allele is not sufficient for transcription.

### Mosaicism between strands of individual DNA molecules

Hairpin-bisulfite PCR enabled us to ask whether or not significant differences in DNA methylation densities exist between the top and bottom strands of individual DNA molecules (Figure 1C). For example, a marked difference in cytosine methylation could arise between the two strands of a DNA molecule if the maintenance DNA methyltransferase, DNMT1, were inhibited during, or immediately after DNA replication [24]. In such cases, an unmethylated bottom (template) strand could be transcribed and contribute to an unexpectedly high level of *FMR1* mRNA. Such strand-specific methylation at transcribed loci has been described for a *globin* gene in chicken embryos [25], and has recently been reported to account for the transcriptional cycling of the *pS2/TFF1* gene [26]. Hairpin-bisulfite PCR can readily detect differences in methylation densities between top and bottom strands of individual molecules [19], and thus enables us to examine the relationship between top- and bottom-strand methylation densities [27].

**Figure 5:**
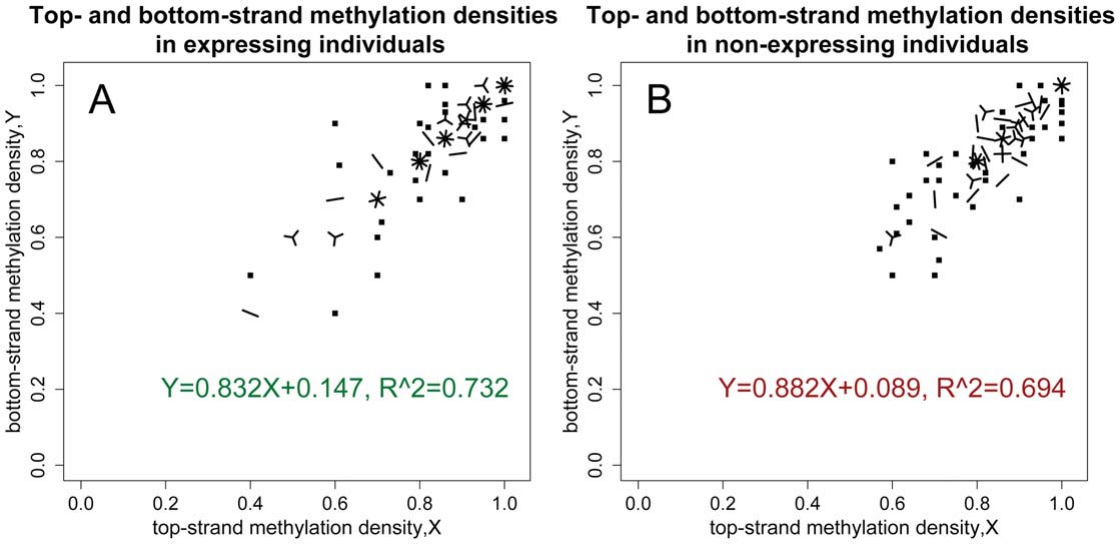
Assessment of methylation mosaicism between the two strands of individual DNA molecules in samples from males with fragile X syndrome. Data are presented separately for samples from *FMR1* mRNA-expressing males (A), and from non-expressing males (B). Weighted linear regression was used to account for differences in the numbers of CpG sites captured by the three different hairpins. The different icons of this “sunflower” plot reflect the number of observed epialleles with a given density of methylation on the top and bottom strands. A single epiallele is represented as a dot, two epialleles with identical methylation densities as a line, three epialleles as a “three-petal” flower, and so on up to the maximum in our data set of nine molecules.

The coding and non-coding strands of double-stranded *FMR1* molecules were highly correlated in their methylation densities for epialleles from both expressing (Figure 5a) and non-expressing (Figure 5b) males. Of particular relevance is our finding that none of the 111 methylated molecules from *FMR1*-expressing individuals had dense methylation on the non-coding, upper strand and low-density methylation on the coding, lower DNA strand (Figure 5a). Thus, we can exclude, with 95% confidence, the existence in *FMR1*-expressing males of a sub-population larger than 3.2% of cells that has markedly discordant methylation patterns between the two strands. This hypothetical 3.2% of cells would need to transcribe *FMR1* to levels more than 30-fold greater than for normal *FMR1* alleles to account for a relative mRNA level that averages 1.0 in these four expressing males. We conclude that methylation mosaicism between top and bottom strands of individual molecules is unlikely to account for *FMR1* expression in these males with FXS.

### Detection of inter-cell mosaicism in *FMR1-*expressing males with full mutation alleles

Because each male cell carries only one X chromosome, the presence of both methylated and unmethylated *FMR1* alleles in DNA isolated from an individual male is evidence of inter-cell mosaicism. For the detection of this type of mosaicism, hairpin I was the most reliable of the three assays. This hairpin assay yielded nearly unbiased amplification of methylated and unmethylated alleles from normal females (Table 1). For the five non-expressing males, all three hairpin assays yielded only methylated epialleles (total = 118). The recovery of 118 methylated epialleles and no unmethylated epialleles excludes with 95% confidence the existence of a subpopulation of unmethylated epialleles that constitutes more than 2.5% of cells in the non-expressing males.

Of the four DNA samples of *FMR1*-expressing males, two showed evidence of inter-cell methylation mosaicism (males #20 and #3; Table 1). DNA from male #3, previously found to have a level of *FMR1* mRNA of 1.4 relative to the mean for normal males [16], had 31% (5/16) unmethylated *FMR1* alleles. DNA from male #20, previously found to have a level of *FMR1* mRNA of 1.0 [16], had 53% (9/17) unmethylated *FMR1* epialleles (Table 1). Because we validated our hairpin-bisulfite PCR products with batch-stamps and barcodes [20], we can be confident that each *FMR1* epiallele we analyzed was amplified from a different genomic template molecule. Likewise, none of our analyzed epialleles, methylated or unmethylated, arose through contamination or template redundancy. For these two males, the proportions of unmethylated epialleles reported here are sufficient to explain the observed levels of *FMR1* mRNA. Cells carrying unmethylated *FMR1* epialleles with expanded CGG repeats are known to transcribe *FMR1* at an increased rate (up to 10-fold) relative to alleles with normal-sized repeats, thereby leading to unusually high expression levels detected by quantitative RT-PCR [23], [28], [29], [30]. It is thus clear that males #3 and #20 are inter-cell methylation mosaics whose levels of *FMR1* mRNA are consistent with their degrees of mosaicism. Such a high level of mosaicism of unmethylated epialleles could have been missed by earlier Southern hybridization if the allele sizes were very heterogeneous.

For two of the expressing males, #78 and #14, no unmethylated epialleles were observed (Table 1). One possible explanation is that for these males, transcription occurred from a densely methylated *FMR1* promoter, as was suggested previously [16]. An alternate possibility is that unmethylated epialleles were present in these individuals, but were not detected. We used a statistical test to assess this possibility. The DNA sample from expressing male #78 gave only seven informative epialleles with hairpin II, all of which were methylated. As described in Materials and Methods, amplification using hairpin II is heavily biased in favor of methylated alleles, and is thus ineffective at revealing low levels of unmethylated alleles. With so few informative epialleles, and such biased amplification of methylated epialleles with hairpin II, we cannot exclude the possibility that the true frequency of unmethylated epialleles in DNA sample #78 exceeds 0.1, which, with overexpression, could account for the observed *FMR1* mRNA level of 0.7 reported for this individual. Nonetheless, we cannot exclude the original proposal [16] that the observed level of *FMR1* RNA in this individual arises from methylated epialleles.

For expressing male #14, the larger number of epialleles available for analysis (85; Table 1), allows a much smaller probability that there is significant but undetected inter-cell mosaicism. In order for the observed mRNA level (0.8) to result from transcription of unmethylated alleles, at least 8–10% of this individual's epialleles would have had to have been unmethylated. The probability that this level of mosaicism existed but was not detected in our study is less than 0.01. Thus, for this individual, inter-cell mosaicism is unlikely to account for the observed levels of *FMR1* RNA.

## Discussion

The different types of mosaicism for DNA methylation can be identified and distinguished with new and very sensitive techniques [19], [20]. New methylation data from some of the DNA samples used by Tassone et al. have allowed us to test for methylation mosaicism among cells, among CpG sites, and between the two strands of DNA. Any one of these three possible forms of methylation mosaicism could, *a priori*, explain unexpected *FMR1* expression in the FXS samples analyzed by Tassone et al. [16].

As described above, we collected double-stranded methylation patterns from DNA of nine males with full mutation alleles reported to be fully methylated, using a subset of the samples that Tassone and colleagues used in their 2001 study [16]. In that study, four of the males with FXS, for which we here report new methylation data, were found to have *FMR1* mRNA levels ranging from 0.7 to 1.4, relative to the mean level for normal controls (1.0) [16]. This result called into question previous reports that methylation of the *FMR1* promoter necessarily results in transcriptional inactivity [13], [31].

One possible explanation for the unexpected finding of Tassone and colleagues is that most research protocols that are designed to ascertain methylation status are not able to detect all possible types of methylation mosaicism. Even for the previously reported inter-cell mosaicism at the *FMR1* locus [3], Southern hybridization does not readily detect mosaicism in those instances where there is a broad size distribution of unmethylated epialleles, and could thus obscure inter-cell mosaicism at *FMR1*. We reasoned that if methylation mosaicism were the basis for the observed *FMR1* expression, then double-stranded DNA methylation patterns of the *FMR1* promoter might reveal mosaicism of an unusual form not previously assessed in FXS. We therefore searched for three possible types of mosaicism using hairpin-bisulfite PCR with batch-stamps and barcodes [19], [20].

Of the three types of methylation mosaicism evaluated in the current study, only inter-cell mosaicism clearly differed between the five non-expressing males with FXS as compared to two of the four *FMR1*-expressing males analyzed. The variations in methylation among CpG sites, and between the coding and non-coding strands of individual DNA duplexes, were similar for expressing and non-expressing males with FXS.

From two of the *FMR1*-expressing males, only methylated alleles were detected. For one of these males (male 78, Table 1), very few alleles were recovered, all of them from a region with a strong bias against amplification of unmethylated alleles, rendering this case uninformative. For this male, neither a high fraction (0.76) of unmethylated, transcriptionally active alleles, nor the possibility that all alleles are indeed methylated, can be excluded. For the other male (male 14, Table 1), numerous epialleles were recovered from all three regions of *FMR1*. After correcting for amplification bias (Text SI), the estimated fraction of unmethylated epialleles is less than 0.01. This hypothetical low level of unmethylated epialleles cannot readily account for the observed mRNA expression (0.8) [16], (Table 1).

The finding of mRNA from *FMR1* in the sample from male #14 thus calls for explanations alternate to the inter-cell methylation-mosaicism hypothesis. It is possible that densely methylated alleles are amenable to transcription under some circumstances, since it is known that hypermethylation *per se* is not sufficient to block transcription. For example, DNA methylated *in vitro* and injected into *Xenopus* oocytes can be transcribed prior to accrual of histones [32], [33]. It is thus conceivable that reduced efficiency of chromatin formation underlies the presence of *FMR1* mRNA in male #14. Additional samples from this individual as well as from others in this sample cohort were not available for further tests of this hypothesis, or indeed, of the formal possibilities of sample mix-up during collection, storage, and/or assays of *FMR1* epialleles and transcript abundances.

To confirm the generality of our findings, it would be useful to analyze additional clinical samples from *FMR1*-expressing fragile X males diagnosed as having only methylated, full mutation epialleles. Such samples, however, are rare. Since the time of the previous publication [16], approximately 200 samples from males with fragile X who have predominantly methylated full mutation epialleles have been received by the Fragile X Patient Recruitment and Evaluation Core at the MIND Institute, University of California, Davis. Of these samples, the majority have *FMR1* mRNA levels less than 0.15 relative to normal males, and none has an mRNA level greater than 0.4. During the same interval, approximately 130 additional samples have been received from fragile X males whose full mutation epialleles had been classified as inter-cell methylation mosaics. This latter group shows considerable variability in *FMR1* mRNA expression, with levels overlapping those reported previously [16]. We have not received any additional samples that both appear fully methylated by Southern analysis, and have high *FMR1* expression. It is possible that development of new methods for detecting methylation mosaicism, such as presented in Dahl et al. [34], and here, has alerted clinical labs to the possibility of cryptic mosaicism, leading to more sensitive classification of methylation status in males with full mutation alleles. Due to this lack of relevant samples, it has been difficult to confirm and extend the finding reported here and elsewhere [34], that cryptic methylation mosaicism can exist in males classified clinically as having only methylated, full mutation *FMR1* epialleles.

The presence of cryptic inter-cell methylation mosaicism in males with full mutations in the *FMR1* gene has implications for the diagnosis and prognosis of FXS. Future clinical and research studies may benefit from assessing the degree of inter-cell mosaicism in conjunction with assays for the levels of *FMR1* mRNA and protein [35]. Measurement of these epigenetic and biochemical parameters may contribute to more accurate prognosis of cognitive function in males with full mutation *FMR1* alleles.

## Materials and Methods

### Ethics statement/Subjects

Written consent was obtained from all participating patients and from female subjects, except for anonymous samples denoted ‘female cF#_’ in Table 1. The consent form was approved by the Institutional Review Board of the University of California at Davis. For “cF#” samples the Human Subjects Division at the University of Washington determined that use of these anonymous samples does not fall under the federal definition of “human subjects research.” Use of these samples is therefore not subject to 45 CFR 46 and does not require review by the Institutional Review Board.

### DNA samples

The nine FXS DNA samples used in this study are a subset of the samples previously described by Tassone and colleagues [16]. We chose to analyze DNA samples from four of the males with FXS who showed significant levels of *FMR1* mRNA expression. The other five FXS DNA samples used here were from males not expressing *FMR1* mRNA (Table 1). DNAs used in control experiments were isolated from peripheral blood leukocytes of twelve females determined to have normal-sized *FMR1* CGG repeats. Four of these DNA samples were provided by F.T. (the M.I.N.D. Institute), and the remaining eight, designated as ‘cF’, were from the C.D.L lab collection. It is not known whether or not skewed X inactivation occurred in any of the female DNA samples. None of the females examined had SNPs that would allow us directly to address the question of activation ratio. Indeed, it is precisely because the nucleotide sequence of the region examined was identical among the subjects examined that data from females can serve as a metric of the bias inherent to a given hairpin assay. Genetic identity at this locus ensures that any deviations from the 50∶50 methylated∶unmethylated ratio expected in data from females can be attributed to the assay itself, rather than to nucleotide-level differences between the two homologs. The recovery bias observed for females, for which the true methylated∶unmethylated ratio is known, can therefore be applied in the interpretation of data from males, for which the true methylated∶unmethylated ratio is unknown.

### Hairpin-bisulfite PCR

For each sample, double-stranded genomic DNA was digested with a restriction enzyme that leaves a single-stranded overhang near the region to be analyzed. A short hairpin oligonucleotide linker was ligated to this overhang at the *FMR1* locus (Figure 2) [19]. This hairpin linker contains a defined “batchstamp” sequence that specifies the date of the reaction and the sample number; a barcode of eight random nucleotides, contained in the “loop” of the hairpin molecule, identifies the original genomic DNA molecule that served as the PCR template [20]. This information is indispensable in assembling validated sets of sequence data that are not corrupted by PCR template contamination or redundancy [20]. Because of the length of the region that we wished to study, three different hairpin linkers and primer sets were necessary to cover the 52 CpG dyads of the *FMR1* promoter (Figure 3).

Protocols were designed to ensure that the CGG repeats of *FMR1* were removed prior to hairpin-linker ligation. The expanded and sometimes hypermethylated repeats in DNAs from patients could affect the denaturability of DNAs during PCR amplification of the *FMR1* promoter region, exacerbating any inherent amplification bias. To eliminate this potential source of bias, our protocol removes the CGG repeat with appropriate restriction enzymes as specified below. Methylated and unmethylated *FMR1* epialleles from patients and control females are thus rendered equivalent at the nucleotide-sequence level for the region analyzed. Hence, it is reasonable to assume, as we do here, that estimates of amplification bias from normal female *FMR1* epialleles can be used to correct for bias in the recovery of expanded *FMR1* epialleles (Text SI).

Following linker ligation, genomic DNA was treated with bisulfite under conditions that convert unmethylated cytosines to uracil with very high efficiency, while leaving methylated cytosines unchanged. After bisulfite conversion, the two strands of linked genomic DNAs are no longer strongly complementary. This reduction in strand complementarity allows conventional PCR amplification of the two now-linked strands of individual DNA molecules, yielding both top- and bottom-strand methylation information in a single sequence read [19].

PCR products were subcloned using the TOPO-TA cloning kit (Invitrogen), and sequenced on an ABI 3100 in the Comparative Genomics Center of the Department of Biology, the University of Washington. Resulting sequences were obtained and scored for CpG-cytosine methylation as described previously [20]. Only molecules with no more than one failure of conversion for non-CpG cytosines were used for this analysis, corresponding to a conversion efficiency exceeding 99%.

The *FMR1* promoter is especially difficult to denature in DNA of individuals with FXS because of augmented levels of DNA methylation [3]. We used ten denaturation steps during the bisulfite conversion reaction to achieve good conversion of this CG-rich promoter. Our protocol for hairpin I has been described previously [19]; in brief, ∼5 ug of DNA was digested with restriction endonucleases DraIII and AluI prior to ligation of the hairpin linkers (5′PAGCGTAGCDDDDDDDDGCTACGCTTGA, where D represents A, G or T, thus generating random barcodes that are not altered by bisulfite conversion). Specified differences in the nonrandom sequences of the hairpin stems, the batch-stamps, create distinct versions of hairpin I for each individual examined. Primers used for hairpin I PCR amplification were: 5′- CCTCTCTCTTCAAATAACCTAAAAAC-3′ (primer 1), and 5′- GTTGYGGGTGTAAATATTGAAATTA-3′ (primer 2), where Y represents C or T. Restriction endonuclease PspGI was used for hairpin II, to cleave the DNA prior to ligation of the hairpin linkers (5′P- CCTGGTGCACGTTDDDDDDDDAACGTGCA-3′, with variations in the hairpin stem as described above. Primers used for PCR amplification with hairpin II were: 5′- CCTAACTAAAACCRAACCCCC-3′, where R represents G or A (primer 1), and 5′- GGAGTTGAGYGTTTGATTAGG-3′ (primer 2).

PCR for hairpins I and II was performed using HotStarTaq MasterMix (Qiagen) and 2% DMSO, with denaturation at 95°C for 15 min., followed by 41 cycles of denaturation at 95°C for 30 sec., annealing at 56°C for 30 sec, and extension at 72°C for 50 sec, followed by a final extension at 72°C for 5 min. For hairpin III, restriction endonuclease PspGI was used to cleave the DNA prior to ligation of the hairpin linkers (5′P- CCAGGAGCGATGCDDDDDDDDGCATCGCT-3′, with variations in the hairpin stem as described above). Primers used for PCR amplification with hairpin III were: 5′- AAAAACRAAACCRAAAAACTAAACCC-3′ (primer 1), and 5′- ATTTGAAGAGAGAGGGYGG-3′ (primer 2). PCR conditions were as described above with annealing at 54°C for 30 sec, and extension at 72°C for 50 sec, followed by a final extension at 72°C for 5 min.

Common sources of error in data from bisulfite-treated DNA include biased PCR amplification of individual genomic templates, and biased cloning of individual PCR products [3]. Both types of bias can lead to inaccurate estimates of the proportions of hypomethylated and hypermethylated epialleles. Our method for estimating the extent of bias in PCR amplification is included in Text SI.

## Supporting Information

Text SI **This document provides additional information on statistical approaches and equations used to calculate the extent of PCR bias for each of our three hairpins.**
(DOC)

## References

[1] Fu YH, Kuhl DP, Pizzuti A, Pieretti M, Sutcliffe JS (1991). Variation of the CGG repeat at the fragile X site results in genetic instability: resolution of the Sherman paradox.. Cell.

[2] McConkie-Rosell A, Lachiewicz AM, Spiridigliozzi GA, Tarleton J, Schoenwald S (1993). Evidence that methylation of the FMR-I locus is responsible for variable phenotypic expression of the fragile X syndrome.. Am J Hum Genet.

[3] Stöger R, Kajimura TM, Brown WT, Laird CD (1997). Epigenetic variation illustrated by DNA methylation patterns of the fragile-X gene FMR1.. Hum Mol Genet.

[4] Amir RE, Van den Veyver IB, Wan M, Tran CQ, Francke U (1999). Rett syndrome is caused by mutations in X-linked MECP2, encoding methyl-CpG-binding protein 2.. Nat Genet.

[5] Braunschweig D, Simcox T, Samaco RC, LaSalle JM (2004). X-Chromosome inactivation ratios affect wild-type MeCP2 expression within mosaic Rett syndrome and Mecp2−/+ mouse brain.. Hum Mol Genet.

[6] Scala E, Longo I, Ottimo F, Speciale C, Sampieri K (2007). MECP2 deletions and genotype-phenotype correlation in Rett syndrome.. Am J Med Genet A.

[7] Hagerman RJ, Rivera SM, Hagerman PJ (2008). The Fragile X Family of Disorders: A Model for Autism and Targeted Treatments.. Current Pediatric Reviews.

[8] Crawford DC, Meadows KL, Newman JL, Taft LF, Scott E (2002). Prevalence of the fragile X syndrome in African-Americans.. Am J Med Genet.

[9] Pesso R, Berkenstadt M, Cuckle H, Gak E, Peleg L (2000). Screening for fragile X syndrome in women of reproductive age.. Prenat Diagn.

[10] Coffee B, Keith K, Albizua I, Malone T, Mowrey J (2009). Incidence of fragile X syndrome by newborn screening for methylated FMR1 DNA.. Am J Hum Genet.

[11] Hagerman PJ (2008). The fragile X prevalence paradox.. J Med Genet.

[12] Bird A (2002). DNA methylation patterns and epigenetic memory.. Genes Dev.

[13] Pieretti M, Zhang FP, Fu YH, Warren ST, Oostra BA (1991). Absence of expression of the FMR-1 gene in fragile X syndrome.. Cell.

[14] de Graaff E, de Vries BB, Willemsen R, van Hemel JO, Mohkamsing S (1996). The fragile X phenotype in a mosaic male with a deletion showing expression of the FMR1 protein in 28% of the cells.. Am J Med Genet.

[15] Mila M, Castellvi-Bel S, Sanchez A, Lazaro C, Villa M (1996). Mosaicism for the fragile X syndrome full mutation and deletions within the CGG repeat of the FMR1 gene.. J Med Genet.

[16] Tassone F, Hagerman RJ, Taylor AK, Hagerman PJ (2001). A majority of fragile X males with methylated, full mutation alleles have significant levels of FMR1 messenger RNA.. J Med Genet.

[17] Feng Y, Zhang F, Lokey LK, Chastain JL, Lakkis L (1995). Translational suppression by trinucleotide repeat expansion at FMR1.. Science.

[18] Primerano B, Tassone F, Hagerman RJ, Hagerman P, Amaldi F (2002). Reduced FMR1 mRNA translation efficiency in fragile X patients with premutations.. RNA.

[19] Laird CD, Pleasant ND, Clark AD, Sneeden JL, Hassan KM (2004). Hairpin-bisulfite PCR: assessing epigenetic methylation patterns on complementary strands of individual DNA molecules.. Proc Natl Acad Sci U S A.

[20] Miner BE, Stöger R, Burden AF, Laird CD, Hansen RS (2004). Molecular barcodes detect redundancy and contamination in hairpin-bisulfite PCR.. Nucleic Acids Res.

[21] Kumari D, Usdin K (2001). Interaction of the transcription factors USF1, USF2, and alpha -Pal/Nrf-1 with the FMR1 promoter. Implications for Fragile X mental retardation syndrome.. J Biol Chem.

[22] Beilina A, Tassone F, Schwartz PH, Sahota P, Hagerman PJ (2004). Redistribution of transcription start sites within the FMR1 promoter region with expansion of the downstream CGG-repeat element.. Hum Mol Genet.

[23] Kenneson A, Zhang F, Hagedorn CH, Warren ST (2001). Reduced FMRP and increased FMR1 transcription is proportionally associated with CGG repeat number in intermediate-length and premutation carriers.. Hum Mol Genet.

[24] Laird C, Jaffe E, Karpen G, Lamb M, Nelson R (1987). Fragile sites in human chromosomes as regions of late-replicating DNA.. TIG.

[25] Singal R, vanWert JM (2001). De novo methylation of an embryonic globin gene during normal development is strand specific and spreads from the proximal transcribed region.. Blood.

[26] Metivier R, Gallais R, Tiffoche C, Le Peron C, Jurkowska RZ (2008). Cyclical DNA methylation of a transcriptionally active promoter.. Nature.

[27] Burden AF, Manley NC, Clark AD, Gartler SM, Laird CD (2005). Hemimethylation and non-CpG methylation levels in a promoter region of human LINE-1 (L1) repeated elements.. J Biol Chem.

[28] Tassone F, Hagerman RJ, Loesch DZ, Lachiewicz A, Taylor AK (2000). Fragile X males with unmethylated, full mutation trinucleotide repeat expansions have elevated levels of FMR1 messenger RNA.. Am J Med Genet.

[29] Tassone F, Hagerman RJ, Taylor AK, Gane LW, Godfrey TE (2000). Elevated levels of FMR1 mRNA in carrier males: a new mechanism of involvement in the fragile-X syndrome.. Am J Hum Genet.

[30] Tassone F, Beilina A, Carosi C, Albertosi S, Bagni C (2007). Elevated FMR1 mRNA in premutation carriers is due to increased transcription.. Rna.

[31] Sutcliffe JS, Nelson DL, Zhang F, Pieretti M, Caskey CT (1992). DNA methylation represses FMR-1 transcription in fragile X syndrome.. Hum Mol Genet.

[32] Kass SU, Landsberger N, Wolffe AP (1997). DNA methylation directs a time-dependent repression of transcription initiation.. Curr Biol.

[33] Sandberg G, Schalling M (1997). Effect of in vitro promoter methylation and CGG repeat expansion on FMR-1 expression.. Nucleic Acids Res.

[34] Dahl C, Gronskov K, Larsen LA, Guldberg P, Brondum-Nielsen K (2007). A homogeneous assay for analysis of FMR1 promoter methylation in patients with fragile X syndrome.. Clin Chem.

[35] Loesch DZ, Huggins RM, Hagerman RJ (2004). Phenotypic variation and FMRP levels in fragile X.. Ment Retard Dev Disabil Res Rev.

