# Supplementary material for: Testing the FMR1 Promoter for Mosaicism in DNA Methylation among CpG Sites, Strands, and Cells in FMR1-Expressing Males with Fragile X Syndrome
Source: PLoS One. 2011 Aug 31;6(8):e23648. doi: 10.1371/journal.pone.0023648 (PMC3166088; doi:10.1371/journal.pone.0023648)
Supplement: Text SI — This document provides additional information on statistical approaches and equations used to calculate the extent of PCR bias for each of our three hairpins. (DOC) [file pone.0023648.s001.doc]

**Supporting Information**

**Testing the *FMR1* promoter for mosaicism in DNA methylation among CpG sites, strands, and cells in *FMR1*-expressing males with fragile X syndrome**

**Reinhard Stöger^1,6^*, Diane P. Genereux^1^, Randi J. Hagerman^3,4^, Paul J. Hagerman^3,5^, Flora Tassone^3,5^, and Charles D. Laird^1,2^***

1 Department of Biology, University of Washington, Seattle WA 98195-0002, USA

2 Department of Genome Sciences, University of Washington, Seattle WA 98195-0002, USA

3 M.I.N.D. Institute, University of California Davis Medical Center, Sacramento, California, USA

4 Department of Pediatrics, University of California, Davis, School of Medicine, Davis, CA, USA

5 Department of Biochemistry and Molecular Medicine, University of California, School of Medicine, Davis, California, USA

6 School of Biosciences, University of Nottingham, UK

***please address correspondence to:**

[Reinhard.Stoger@nottingham.ac.uk](mailto:Reinhard.Stoger@nottingham.ac.uk) or cdlaird@u.washington.edu

Reinhard Stöger

School of Biosciences

University of Nottingham

Sutton Bonington Campus

Leicestershire

LE12 5RD

UK

Tel: ++ 44(0)115 951 6232

FAX: ++ 44(0)115 951 6302

**Estimating the extent of bias in PCR amplification**

1. Because each hairpin is an independent experiment, the extent of bias due to PCR and cloning may differ among hairpins. Female DNAs provide an indicator of whether or not a given hairpin yields amplification bias, as well as quantitative information that enables reliable data analysis even when bias is severe [1]. Human females have one active-X and one inactive-X chromosome in each somatic cell. Normal *FMR1* alleles on the active-X chromosome lack methylation; by contrast, normal *FMR1* alleles on the inactive X are methylated at the majority of CpG sites (> 60%) [1,2] (Table 1). Unbiased amplification and cloning of sequences from *FMR1* alleles from normal females should yield data sets comprised of equal proportions of methylated and unmethylated epialleles. The extent of deviation from this expected 1:1 ratio in sequences recovered from a normal female thus provides an index of the recovery bias inherent to a given hairpin. Therefore, we can use the hairpin-specific recovery bias in data from female DNA to improve the accuracy of our analysis of data from males with fragile X.

The extent of bias in data from female DNAs differed among the three hairpins (Table 1). Hairpin I (CpG sites 1 – 22) showed only modest skewing of PCR products with approximately 0.45 (175 of 387) of recovered epialleles observed to be methylated. Hairpin III showed a more pronounced bias in the opposite direction: 0.66 (55 of 83) of recovered epialleles were methylated. Hairpin II produced very skewed results, yielding 0.92 of methylated epialleles (72 of 78). Our experience and that of others indicates that biased amplification of bisulfite-based PCR products is common [1]; we have not explored the reasons for this bias. Nevertheless, the statistical method we describe here enables us to deal with these biases and to make useful inferences from all three hairpins.

The observed frequencies of methylated, inactive-X epialleles, *o*_m_, are related to their true frequencies by

[Equation 1]

where *m* indicates the true overall frequency of methylated epialleles, and *b*_h_ gives the probability of sampling a methylated allele using hairpin *h*. A *b_h_* value of 0.5 would indicate a perfect, unbiased sampling procedure; values of greater than 0.5 would indicate oversampling of methylated epialleles; and values of less than 0.5 would indicate undersampling. We can then write a binomial function for the likelihood, *L*, of observing a given pair of methylated, *M*, and unmethylated, *U*, epiallele counts as a function of the recovery bias, *b_h_*, and of the true frequency of methylated epialleles, *m*.

[Equation 2]

We set *m* = 0.5 for the normal-female data, and maximized the likelihood of this function to obtain a point estimate of *b_h_* for each hairpin. We used the method of Meeker and Escobar [3] to calculate 95% confidence intervals on the recovery bias for the normal-female data reported in Table 1. These confidence intervals were (0.40, 0.50), (0.84, 0.97) and (0.55, 0.76), for hairpins I, II, and III, respectively.

Data from each of the three hairpins contain information on the overall frequency of methylated epialleles in a given DNA sample. Equation 3 incorporates information from all three hairpins to give the upper bound on the frequency of unmethylated epialleles from *FMR1*-expressing males with fragile X syndrome. Briefly, this function finds the product of equation 2 calculated for hairpins 1-3. The denominator integrates over *m* values from 0 to 1 – that is, over all possible values for the methylation frequencies ascertained for each of the three hairpins. The numerator integrates over *m* values from *z* to 1. We solve to find *z*, the value of *m* that results in integration over 95% of the mass of this distribution. With 95% probability, the frequency of methylated epialleles. from a male with fragile X is *z* or larger. Thus, these equations allow us to identify the upper bound on the frequency of unmethylated epialleles from a male with fragile X, given the observed data.

[Equation 3]

**References**

1. Stöger R, Kajimura TM, Brown WT, Laird CD (1997) Epigenetic variation illustrated by DNA methylation patterns of the fragile-X gene FMR1. Hum Mol Genet 6: 1791-1801.

2. Hansen RS, Gartler SM, Scott CR, Chen SH, Laird CD (1992) Methylation analysis of CGG sites in the CpG island of the human FMR1 gene. Hum Mol Genet 1: 571-578.

3. Meeker WQ, Escobar LA (1995) Teaching about approximate confidence regions based on maximum likelihood estimation. Am Stat 49: 48-53.
